# Efficacy and safety of hepatic arterial infusion chemotherapy combined with lenvatinib and PD-1 inhibitors for advanced hepatocellular carcinoma with macrovascular invasion

**DOI:** 10.1186/s12957-024-03396-4

**Published:** 2024-05-06

**Authors:** Yufeng Zhang, Haiyan Zhang, Haoqian Xu, Ying Wang, Long Feng, Fengming Yi

**Affiliations:** 1 Department of Oncology, Second Affiliated Hospital of Nanchang University, Nanchang, 330006 P.R. of China; 2 Jiangxi Key Laboratory of Clinical and Translational Cancer Research, Nanchang, 330006 P.R. of China

**Keywords:** Hepatocellular carcinoma, Macrovascular invasion, Hepatic arterial infusion chemotherapy, Lenvatinib, Programmed cell death-1 inhibitor

## Abstract

**Background and aims:**

The prognosis of hepatocellular carcinoma (HCC) with macrovascular invasion(MaVI)is poor, and the treatment is limited. This study aims to explore the efficacy and safety of hepatic arterial infusion chemotherapy (HAIC), combined with lenvatinib and programmed cell death-1(PD-1) inhibitor in the first-line treatment of HCC with MaVI.

**Methods:**

From July 2020 to February 2022, we retrospectively analyzed consecutive patients with HCC with MaVI who received hepatic arterial infusion FOLFOX(oxaliplatin, 5-fluorouracil, and leucovorin)combined with lenvatinib and PD-1 inhibitor. The efficacy was evaluated by RECIST 1.1. Kaplan-Meier was used to explore the overall survival and progression-free survival (PFS), and the COX regression model was used to analyze the risk factors of PFS. Adverse events (AEs) were evaluated according to CTCAE5.0.

**Results:**

Thirty-two patients with HCC complicated with MaVI were recruited from the Second Affiliated Hospital of Nanchang University. Among the patients treated with HAIC combined with lenvatinib and PD-1 inhibitor, ten patients (31.25%) got partial response, eighteen patients (56.25%) maintained stable disease and four patients (12.50%) suffered progressive disease during follow-up; and objective response rate was 31.25%, and disease control rate was 87.5%. The median PFS was 179 days. Univariate and multivariate Cox analysis showed that the extrahepatic metastases and Child-Pugh score were independent prognostic factors of PFS. Twenty-two (68.75%) patients suffered adverse reactions. The main AEs were elevated transaminase (46.87%), thrombocytopenia (40.63%), hypoalbuminemia (28.13%), nausea and vomiting (21.88%), leukopenia (18.76%), abdominal pain (15.63%), hypertension (15.63%) and fever (15.63%). There were seven cases (21.88%) that had grade 3 or above AEs; Among them, two cases with elevated transaminase (6.25%), leukopenia, thrombocytopenia, nausea and vomiting, abdominal pain, and diarrhea occurred in one case respectively. Moreover, no treatment-related death was observed.

**Conclusions:**

Hepatic arterial infusion of FOLFOX combined with lenvatinib and PD-1 inhibitor as the first-line treatment for HCC complicated with MaVI is effective, and adverse reactions are tolerable.

## Introduction

Primary liver cancer is the sixth highest morbidity cancer in the world and the third highest cause of cancer mortality. China has the largest population of primary liver cancer. An estimated 9.5 and 8.7 new cases and deaths of primary liver cancer, respectively, per 100,000 people globally in 2020. Hepatocellular carcinoma (HCC) is the dominate subtype of primary liver cancer, accounting for nearly 80% of the total population [1–3]. HCC has high morbidity and mortality worldwide [4], as 50% of patients are diagnosed at an advanced stage with vascular invasion or distant metastasis [5]. The most common macrovascular invasive include portal vein and/or hepatic vein, with an incidence of 10-40% [6]. Portal vein invasive forms portal vein tumor thrombus (PVTT) [7]; Outflow tract invasive including hepatic vein tumor thrombus (HVTT), inferior vena cava tumor thrombus (IVCTT), and right atrium tumor thrombus (RATT) [8–10]. PVTT in HCC will induce intrahepatic and extrahepatic metastasis within a short time; and variceal bleeding, hepatic encephalopathy, liver failure, or other fatal sequelae due to the portal hypertension caused by tumor thrombus. HVTT or IVCTT may lead to pulmonary embolism and cardiac tamponade. These patients have a poor prognosis, with a median survival time of only 2.7 to 4.0 months without treatment [11, 12].

Sorafenib has been the only first-line treatment in HCC patients with vascular invasive for a long time [13, 14]. Subsequently, Lenvatinib was introduced to these patients in 2018, with a higher objective response rate (ORR) and median progression-free survival(mPFS) (18.8% vs. 6.5%; 7.4 months vs. 3.7 months) [15–19]. In recent years, Immune checkpoint inhibitors including programmed cell death-1 (PD-1) inhibitors and programmed cell death-Ligand (PD-1) 1, PD-L1) inhibitors have achieved good clinical efficacy and safety in patients with advanced HCC [20]. In 2020, the IMbrave 150 study “T + A” regimen (atezolizumab plus bevacizumab), which increased the ORR to 27.3%, was approved as the first-line treatment for advanced liver cancer [21]. At the same time, phase Ib Study Keynote 524 indicated that lenvatinib plus pembrolizumab increased the ORR to 36% [22].

Apart from systematic therapy, locoregional treatment has already been confirmed to increase efficacy in patients with advanced HCC. For example, a study enrolled 262 patients with 65.6% macrovascular invasion comparing hepatic arterial infusion chemotherapy (HAIC) and sorafenib demonstrated HAIC achieved better overall survival (OS) than Sorafenib (13.9 months vs. 8.2 months) [23]. Another clinical trial that enrolled 247 HCC patients with portal vein invasion indicated that sorafenib plus HAIC has significantly longer OS when compared with sorafenib monotherapy (13.37 months vs. 7.13 months) [24]. More and more studies are trying to explore the safety and efficacy of the combination of HAIC, anti-PD-1/PD-L1 immunotherapy, and molecularly targeted agents and find the promising benefits of these combinations [25, 26]. However, no study focuses on HCC patients with macrovascular invasion. Herein, we conducted the current study to investigate the efficacy and of HAIC combined with lenvatinib and PD-1 immunotherapy in the first-line treatment of patients with advanced hepatocellular carcinoma with macrovascular invasion.

## Methods

General Information: Thirty-two HCC patients with macrovascular invasive who received HAIC combined with lenvatinib and PD-1 inhibitor as first-line treatment were retrospectively enrolled in the second affiliated hospital of Nanchang University from July 2020 to February 2022, including 24 patients with portal vein tumor thrombus and 8 patients with hepatic vein and inferior vena cava tumor thrombus. The inclusion criteria for this study were as follows: aged was between 18 and 75 years old; Child-Pugh liver function was ≤ 7; pathologically confirmed hepatocellular carcinoma or met the clinical diagnostic criteria HCC [27]; Macrovascular invasion, and the Japanese classification of macrovascular invasion was used in this study [28, 29]; Advanced stage of liver cancer that is inoperable; No other treatment was received at initial diagnosis; Eastern Cooperative Oncology Group (ECOG) lower than 2; No history of autoimmune diseases; Willingness to adopt the treatment regimen and signed the informed consent. The exclusion criteria are as follows: Hepatic decompensation, such as hepatic encephalopathy, severe ascites, gastroesophageal variceal bleeding, coagulopathy, and/or combined bleeding tendency; Patients with a previous or concurrent history of other malignant tumors.

Treatment: Arterial infusion mFOLFOX6 regimen: oxaliplatin 85 mg/m2 for 2 h; Calcium folinate 400 mg/m2 was given by arterial infusion for 2 h; Bolus injection of 5-fluorouracil 400 mg/m2, followed by arterial infusion of 2400 mg/m2 for 46 h; 3 weeks as a cycle. Lenvatinib was administered orally at a standard dose (12 mg for body weight ≥ 60 kg and 8 mg for body weight < 60 kg) once daily. PD-1 antibodies including camrelizumab, sintilimab, and tislelizumab were injected every 3 weeks. An imaging examination was routinely performed every 6 weeks. Tumor response was evaluated according to RECIST1.1. Adverse reactions were assessed according to Common Terminology Criteria for Adverse Events 4.0 (CTCAE4.0).

Statistical Analysis: SPSS 26.0 was used for data sorting and statistical analysis. PFS was analyzed by Kaplan-Meier, and the log-rank test was used to analyze the differences in survival curves. Univariate and multivariate Cox regression models were used to analyze the clinical factors that affect PFS. *p* < 0.05 indicated a significant difference.

## Results

### Patient characteristics

A total of 32 patients with HCC from July 2020 to February 2022 were enrolled in this study, including 27 males and 5 females. Seventeen patients were ≥ 50 years old, and 15 patients <50 years old; All of the patients has the history of hepatitis; Twenty one patients were complicated cirrhosis; The AFP values of twenty three patients were ≥ 400 ng/mL, and nine patients were <400 ng/mL; Sixteen patients were complicated with portal hypertension; All patients were complicated with macrovascular invasion, including nine cases of Vp1-3, fifteen cases of Vp4, and eight cases of Vv1-3(Vp1 indicates the presence of a tumor thrombus distal to the second-order branches of the portal vein (but no direct involvement); Vp2 is invasion of the second order branches of the portal vein; Vp3 is the presence of the tumor thrombus in the first-order branch; Vp4 includes tumor thrombus in the main trunk of the portal vein or a portal vein branch contralateral to the primarily involved lobe. Vv1 represents tumor thrombus in a peripheral hepatic vein; Vv2 indicates a major hepatic vein involvement, and Vv3 indicate the inferior vena cava invasion).; twenty three patients had multiple intrahepatic lesions and nine had single lesions; thirteen patients had tumor size greater than 10 cm; Four patients have extrahepatic spread; Five patients were ECOG score 0, and twenty seven patients were ECOG score 1; Twenty eight patients were Child-Pugh A, and four patients were Child-Pugh B; Twenty eight patients get antiviral treatment. The clinical features are shown in Table 1.

**Table 1:** Clinical features of patients enrolled

Characteristics	*n*	(%)
Sex		
male	27	84.4
female	5	15.6
Age		
≥50 years old	17	53.1
<50 years old	15	46.9
History of hepatitis		
yes	32	100
no	0	0
History of cirrhosis		
yes	21	65.6
no	11	34.4
Baseline AFP values		
≥ 400 ng/mL	23	71.9
<400 ng/mL	9	28.1
Complicated with portal hypertension		
yes	16	50.0
no	16	50.0
Classification of tumor thrombus		
Vp1-3	9	28.1
Vp4	15	46.9
Vv1-3	8	25.0
Number of tumors		
multiple	23	71.9
single	9	28.1
Tumor size		
≥10 cm	13	40.6
<10 cm	19	59.4
Extrahepatic metastasis		
yes	4	12.5
no	28	87.5
ECOG score		
0	5	15.6
1	27	84.4
Child-Pugh		
A	28	87.5
B	4	12.5
Antiviral therapy		
yes	28	87.5
no	4	12.5

**Fig. 1:**
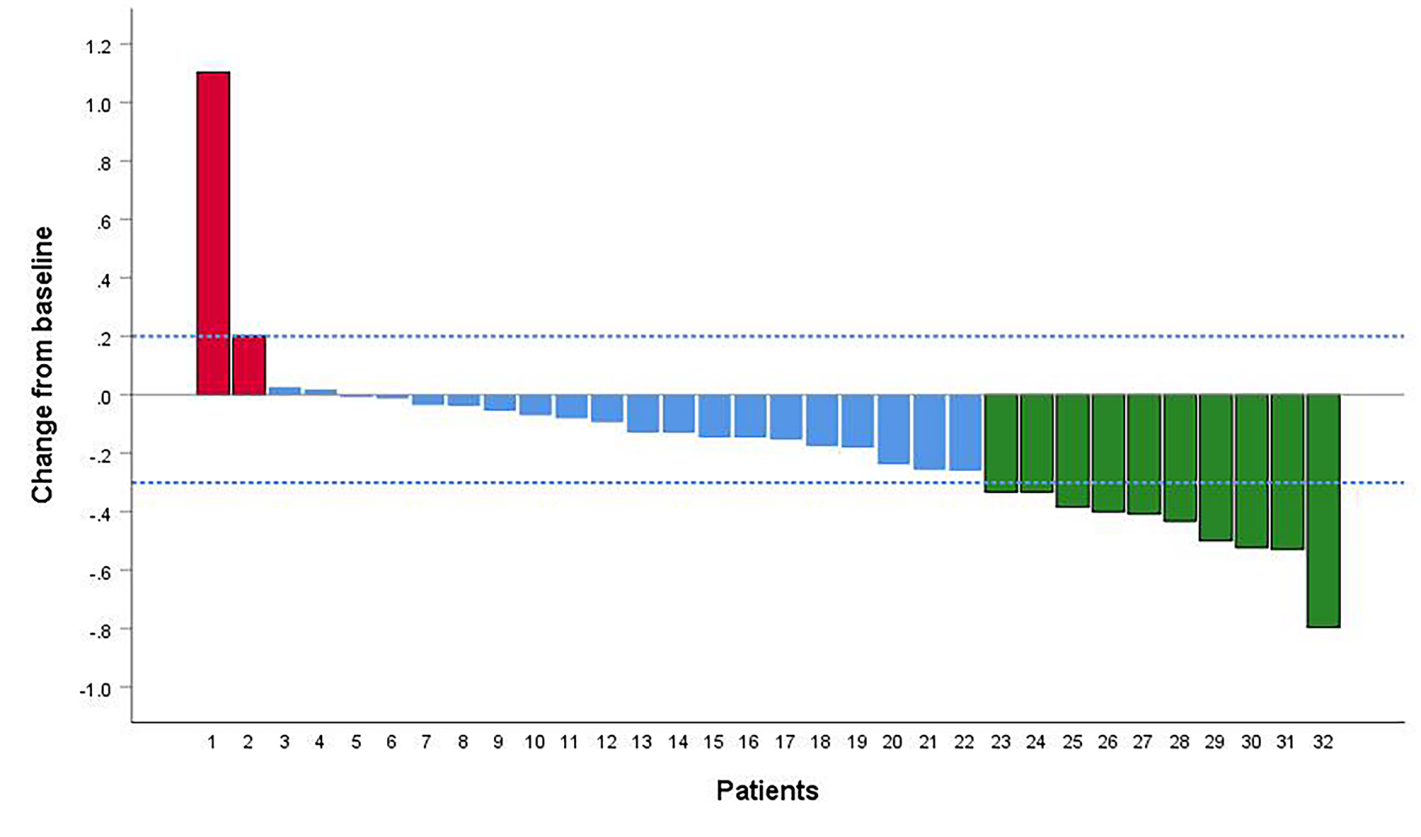
Efficacy of Hepatocellular carcinoma patients with macrovascular invasion received hepatic arterial infusion chemotherapy combined with Lenvatinib and programmed cell death-1 monoclonal antibody. Red bar indicates progressive disease; Blue bar indicates stable disease, Green bar indicates partial response

**Efficacy**: According to RECIST1.1 [30], 10 patients (31.25%) achieved partial response, 18 patients (56.25%) achieved stable disease, and 4 patients (12.50%) suffered progressive disease after 2 cycles. The ORR and DCR were 31.25% and 87.50%, respectively. The evaluation of intrahepatic lesions: 10 patients (31.25%) achieved partial response, 20 patients (62.50%) achieved stable disease, and 2 patients (6.25%) suffered progressive disease. The ORR and DCR were 31.25% and 93.75%, respectively. The details are demonstrated in Table 2; Fig. 1.

**Table 2:** The efficacy of triple therapy for patients enrolled

Evaluation	*n*	%
Overall tumor evaluation		
CR	0	0
PR	10	31.25
SD	18	56.25
PD	4	12.5
ORR	10	31.25
DCR	28	87.5
Evaluation of intrahepatic lesions		
CR	0	0
PR	10	31.25
SD	20	62.5
PD	2	6.25
ORR	10	31.25
DCR	30	93.75

CR: complete response; PR: partial response; SD: stable disease; PD: progressive disease;

DCR: disease control rate; ORR: objective response rate

### Details of treatment

The 32 patients in the study had received 87 cycles of HAIC (median, 2.7 cycles), 147 cycles of immunotherapy (85 cycles of camrelizumab, 26 cycles of sintilimab, and 36 cycles of tislelizumab), and a median duration of lenvatinib treatment for 3.4 months before censored. In the follow-up treatment, three patients underwent radical resection, therefore the conversion surgery rate was 9.38%; Criteria for resectability after conversion therapy included [1] ECOG 0–1, a Child–Pugh score ≤ 7 points; [2] patients with liver cirrhosis having a remnant liver volume ≥ 40% of standard liver volume, or patients without liver cirrhosis having a remnant liver volume ≥ 30% of standard liver volume; [3] magnetic resonance imaging (MRI) indicating inactivation and regression of vascular tumor thrombi, surgical margin ≥ 1.0 cm; and [4] no other contraindications for surgery. Three patients received radiotherapy for vascular invasion, including portal vein tumor thrombus (*n* = 1) and hepatic vein and vena cava tumor thrombus (*n* = 2). Five patients stopped HAIC after achieving PR and only used lenvatinib combined with PD-1 inhibitor as maintenance therapy. One patient was treated with regorafenib combined with sintilimab as a second-line treatment. Best supportive care was used in 2 patients.

**Fig. 2:**
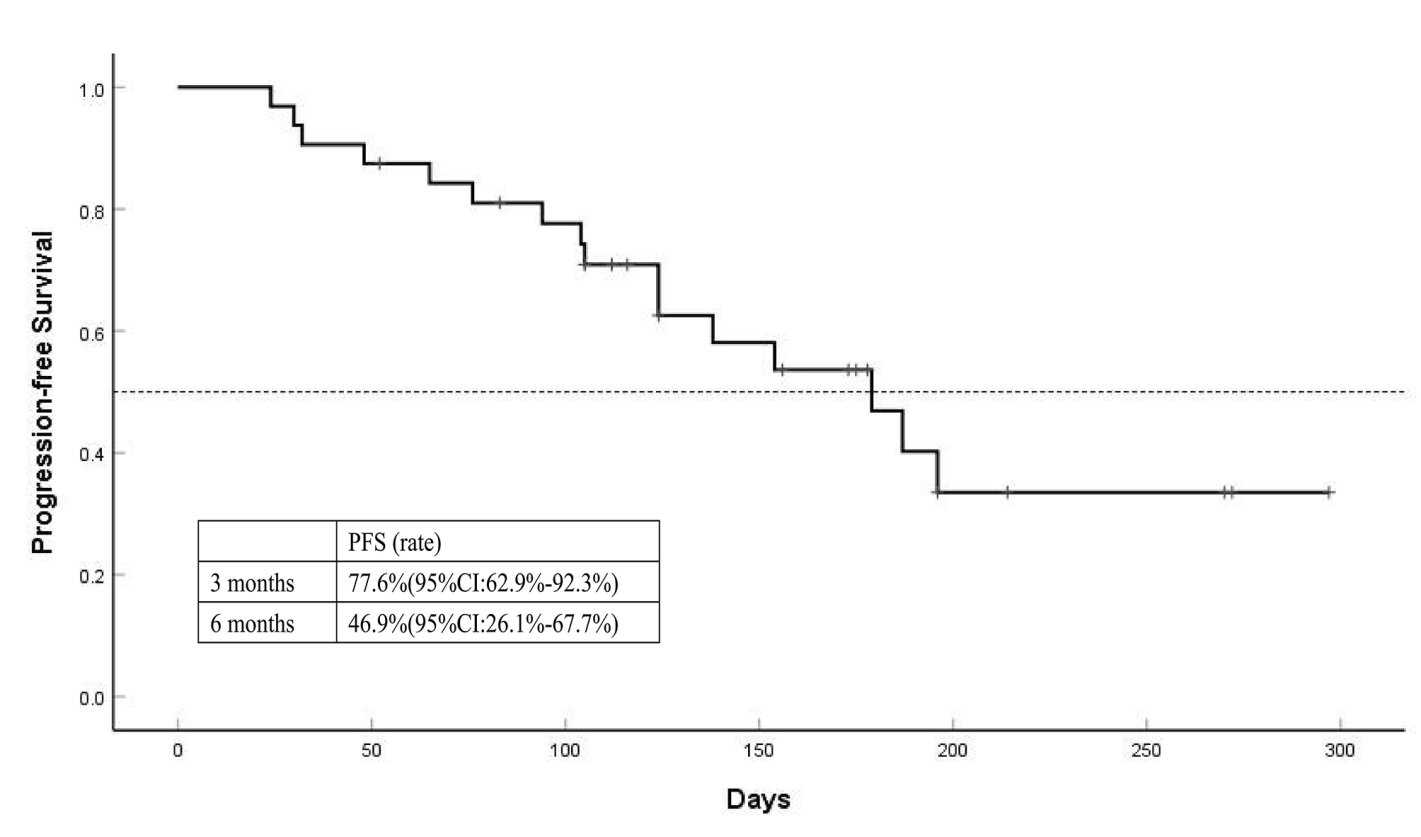
Progression-free survival of patients enrolled. The median progression-free survival of the 32 patients was 179 days (95%CI: 122–236). The 3-month and 6-month progression-free survival rates were 77.6% (95%CI: 62.9-92.3%) and 46.9% (95%CI: 26.1-67.7%)

**Fig. 3:**
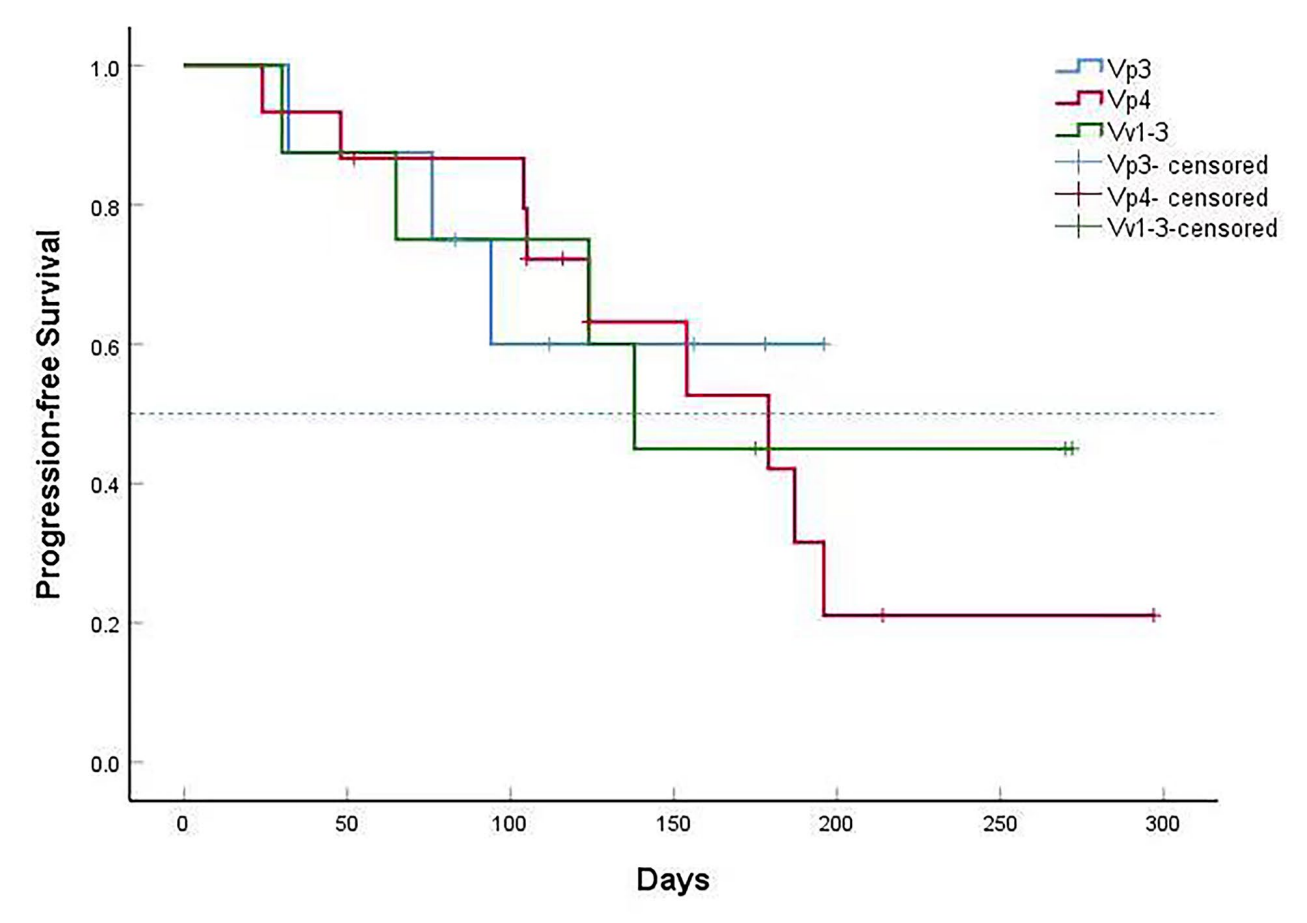
Progression-free survival in subgroups. The median progression-free survival was not reached in patients with Vp3 tumor thrombus, 179 days in patients with Vp4 tumor thrombus, and 138 days in patients with Vv1-3 tumor thrombus (*P* = 0.905). There was no statistically significant difference in PFS between different tumor thrombus grades

**Fig. 4:**
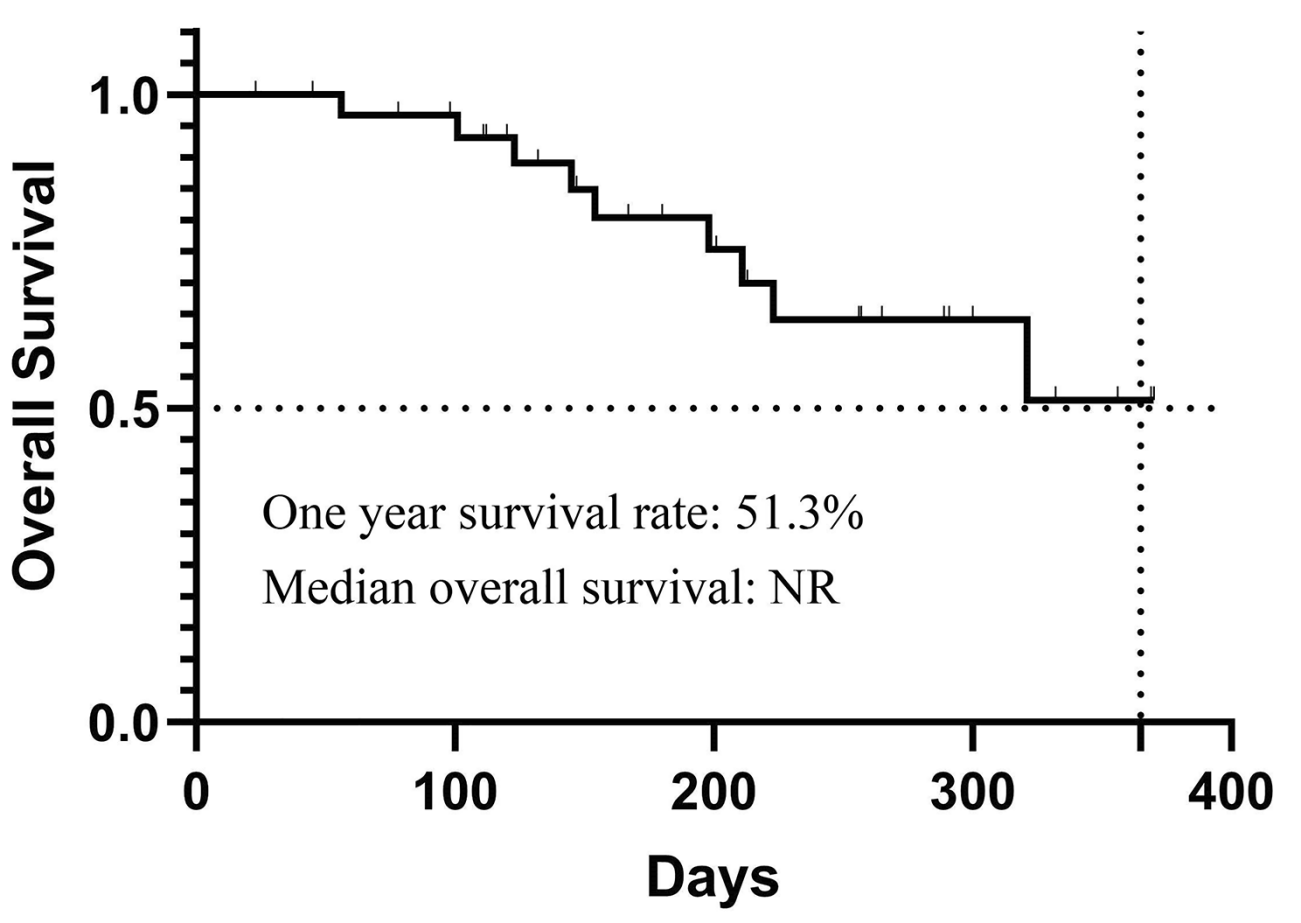
Overall survival of patients enrolled. The median overall survival of the 32 patients was not reached. The one-year survival rate was 51.3%

**Survival Analysis**: By the date of censoring, progression had been observed in 16 patients (50.00%). The median PFS of the 32 patients was 179 days (95%CI: 122–236). The 3-month and 6-month PFS rates were 77.6% (95%CI: 62.9-92.3%) and 46.9% (95%CI: 26.1-67.7%). The results are presented in Fig. 2. The PFS of different thrombus grades were analyzed. The median PFS was not reached in patients with Vp3 tumor thrombus, 179 days in patients with Vp4 tumor thrombus, and 138 days in patients with Vv1-3 tumor thrombus (*P* = 0.905). There was no statistically significant difference in PFS between different tumor thrombus grades. The results are shown in Fig. 3. The median OS was not reached in our study, and one-year survival rate was 51.3%. The result was shown in Fig. 4.

**Fig. 5:**
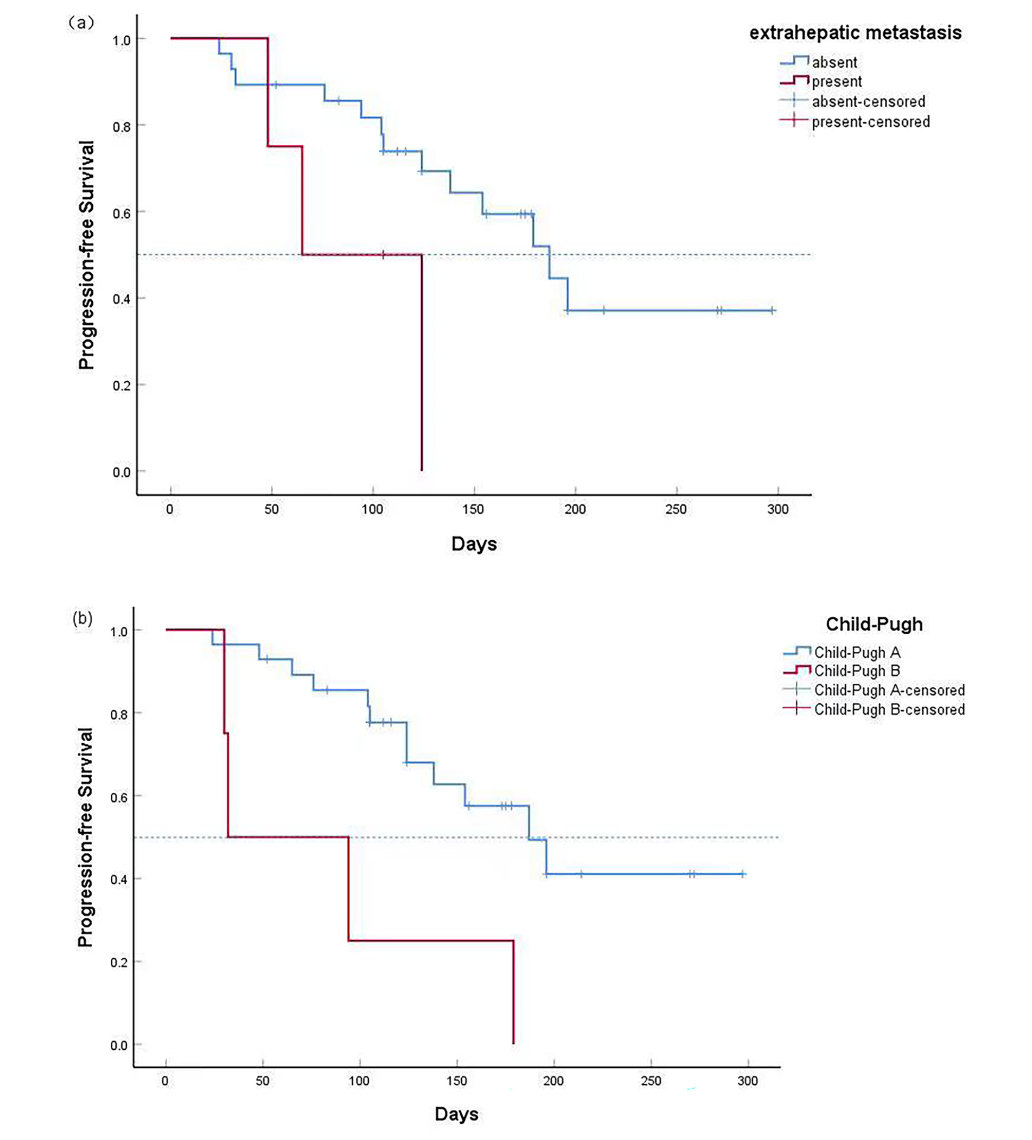
Kaplan-Meier curves for progression-free survival. (**a**)Progression-free survival in patients with and without extrahepatic metastasis; The risk of disease progression in patients with extrahepatic metastasis was higher than that in patients without extrahepatic metastasis (HR = 5.036, 95%CI:1.228–20.653);(**b**) Progression-free survival in patients with Child-Pugh grade A and Child-Pugh grade B. The risk of disease progression in patients with Child-Pugh B was higher than that in patients with Child-Pugh A (HR = 0.163). (95%CI: 0.047–0.564)

**Univariate analysis and multivariate analysis**: Univariate analysis and multivariate Cox regression analysis were used to analyze the clinical characteristics that may affect PFS. The results showed that extrahepatic metastasis and Child-Pugh score were independent prognostic factors for PFS. The risk of disease progression in patients with extrahepatic metastasis was higher than that in patients without extrahepatic metastasis (HR = 5.036, 95%CI:1.228–20.653). The risk of disease progression in patients with Child-Pugh B was higher than that in patients with Child-Pugh A (HR = 0.163). (95%CI: 0.047–0.564). The results are shown in Table 3; Fig. 5.

**Table 3:** Univariate analysis and COX regression multivariate analysis of progression-free survival in patients enrolled

Group	Univariate Analysis		Multivariate Analysis	
	HR(95%CI)	*P*	HR(95%CI)	*P*
Age(≥50 /<50)	0.938(0.344–2.558)	0.901		
History of cirrhosis (yes/no)	0.496(0.185–1.326)	0.162		
AFP value(>400ng/mL/<400ng/mL)	0.947(0.336–2.669)	0.918		
Combined with portal hypertension (yes/no)	0.620(0.225–1.707)	0.355		
Classification of tumor thrombus	1.093(0.548–2.179)	0.802		
Number of tumors (multiple/single)	0.920(0.315–2.685)	0.878		
Tumor size(>10cm/≤10cm)	1.666(0.618–4.490)	0.313		
Extrahepatic metastasis (yes/no)	3.462(0.906–13.225)	0.069	5.036(1.228–20.653)	0.025
ECOG score (0/1)	1.850(0.417–8.207)	0.418		
Child-Pugh(A/B)	0.215(0.066–0.696)	0.010	0.163(0.047–0.564)	0.004

Safety: Adverse reactions were evaluated according to CTCAE5.0(https://ctep.cancer.gov/protocoldevelopment/electronic_applications/ctc.htm). Among the 32 patients, 22 patients (68.75%) had adverse reactions. The main adverse reactions were elevated transaminase (46.87%), thrombocytopenia (40.63%), hypoalbuminemia (28.13%), nausea and vomiting (21.88%), leukopenia (18.76%), abdominal pain (15.63%), hypertension (15.63%), and fever (15.63%). Seven patients (21.88%) had grade 3 or above adverse reactions, including two patients (6.25%) with elevated transaminase, one patient with leukopenia, one patient with thrombocytopenia, one patient with nausea and vomiting, one patient with abdominal pain and one patient with diarrhea. The dose of lenvatinib was reduced as elevated transaminase, leukopenia, thrombocytopenia, and diarrhea in five patients. No treatment-related death was observed. The results are shown in Table 4.

**Table 4:** The adverse reactions of patients who received triple therapy

Adverse reactions	classification					%	
	1	2	3	4		Grade 1–2	Grades 3–4
Leukopenia	3	2	1	0		15.63	3.13
Thrombocytopenia Elevated aminotransferase Elevated bilirubin Decreased albumin Hypertension Abdominal pain Nausea and vomiting	7 6 4 5 3 2 1	5 7 1 4 2 2 5	1 2 0 0 0 1 1	0 0 0 0 0 0 0		37.5 40.62 15.63 28.13 15.63 12.5 18.75	3.13 6.25 0 0 0 3.13 3.13
Hypothyroidism	1	2	0	0		9.38	0
Fever	2	3	0	0		15.63	0
Diarrhea	2	2	1	0		12.5	3.13

## Discussion

The incidence and mortality of HCC are rising worldwide [4]. Although progress has been made in the diagnosis and treatment of HCC in recent years, the prognosis of patients with advanced HCC is still very poor [31, 32], especially complicated by vascular invasion [33].

At present, there is no consensus on the diagnosis and treatment standards of HCC patients with vascular invasion in the world. According to the BCLC staging, they are classified as advanced stage (stage C), and the treatment strategy is mainly systemic therapy [34]. Systemic therapy includes targeted therapy, immunotherapy, chemotherapy, etc. The first-line systemic therapy recommended by current guidelines for advanced liver cancer includes sorafenib, lenvatinib, and atezolizumab combined with bevacizumab, among which the combination of targeted therapy and immunotherapy has the best efficacy [35].

Locoregional therapy has been validated to improve the effectiveness of monotherapy of systematic treatment for advanced HCC [24, 26, 36, 37]. For locoregional therapy, TACE and HAIC are the main options for advanced HCC [38]. However, HAIC is superior to TACE for patients with large HCC or unresectable [39–42]. A study demonstrated that HAIC has better efficacy than TACE for patients with PVTT, with a median OS of 20.8 months in the HAIC group and 4.0 months in the TACE group (*P* < 0.001). Moreover, fewer adverse reactions were observed in the HAIC group [43]. In this study, all the patients received HAIC with lenvatinib and a PD-1 inhibitor. Median progression-free survival was 179 days. According to the RECIST 1.1 criteria, among the 32 patients, 10 (31.25%) achieved PR, 18 (56.25%) achieved SD, and 4 (12.50%) had PD. The ORR and DCR were 31.25% and 87.50%, respectively. In addition, the safety profile was manageable. In multivariate analysis, extrahepatic metastasis and Child-Pugh classification were independent prognostic factors for PFS. The results were worse than in previous studies [24, 25]. The main reasons for the difference may be the following: the current study not only included HCC with PVTT, but also patients with hepatic vein and vena cava tumor thrombus, which accounted for 25%, and the average HAIC cycles received in this study was less than that of the studies before. All these reasons may lead to a slightly worse PFS in this study. The ORR of patients receiving HAIC, lenvatinib, and PD-1 inhibitor triple therapy in this study (31.25%) may be better than that of patients receiving other first-line systemic treatment [13, 19, 21, 44, 45]. The current standard treatments for HCC with macrovascular invasion including sorafenib, lenvatinib, and atezolizumab plus bevacizumab. The objective response rates were 2%, 24.1%, and 27.3% for sorafenib, lenvatinib, and atezolizumab plus bevacizumab group; Progression-free survival for sorafenib was 7.4 months, and 6.8 months for Atezolizumab plus Bevacizumab; The median overall survival was 10.7 months for sorafenib, 13.6 months for lenvatinib, 19.2 for Atezolizumab plus bevacizumab [13, 19, 21, 46]. However, the main subgroup in a study was Vp4 PVTT, the PFS was 4.9 months, the OS was 9.4 months, and the objective response rate of PVTT based on mRECIST was 61.5% who received PD-1 inhibitor lenvatinib plus radiotherapy in previous study [47]; The intrahepatic tumor objective response rate was 68.3%, and the median OS, PFS was 21.7 months and 14.5 months for patients with main trunk portal vein tumor thrombus who received transarterial chemoembolization plus lenvatinib and PD-1 inhibitors [48]. The high ORR observed in patients receiving HAIC, lenvatinib, and PD-1 antibodies may be due to the synergistic antitumor effects of HAIC, lenvatinib, and PD-1 inhibitors. Locoregional chemotherapy may activate the adaptive immune system by increasing human leukocyte antigen expression and enhancing T cell stimulation [49], and help restore immune surveillance by interfering with signal transduction and transcriptional activator 6-mediated immunosuppression [50]. In addition, chemotherapy can increase antigenicity by inducing immunogenic cell death of tumor cells as well as reducing “off-target” immunosuppression in the tumor microenvironment [51]. As for conversion success rate, the conversion rate in current study was lower than the research reported [52–56] owing to the patients that enrolled in this study were mostly PVTT vp4, HVTT or IVCTT, and the patients in the previous studies were in earlier stage or limited to portal vein invasion Vp1-3. In this study, 9 patients were portal vein invasion Vp1-3, and 3 patients got radical resection after the combination therapy, the conversion success rate in this group is much the same as the studies reported before. In univariate and COX regression multivariate analyses, extrahepatic metastasis and Child-Pugh classification were independent prognostic factors for PFS. The risk of disease progression in patients with extrahepatic metastasis was higher than that in patients without extrahepatic metastasis, and the risk of disease progression in patients with Child-Pugh class B was higher than that in patients with Child-Pugh class A. However, the number of patients with extrahepatic metastasis and Child-Pugh class B was small (*n* = 4), and the results of this study need to be further explored. In addition, we also analyzed different tumor thrombus grades, and the results showed that mPFS of Vp1-3 was not reached, Vp4 was 179 days, and Vv1-3 was 138 days (*P* > 0.05). There was no significant difference. The multivariate analysis of a previous study in 2019 showed that the Vp grade of PVTT was an independent prognostic factor [24]. However, in this study, no relevant results were obtained probably because of the small sample size enrolled in this study.

The adverse reactions of hepatic arterial infusion chemotherapy mainly include upper abdominal pain caused by continuous arterial infusion of chemotherapeutic agents and adverse reactions caused by chemotherapy drugs (including leucopenia, thrombocytopenia, liver function damage, fever, nausea, and vomiting, etc.), but these adverse reactions are milder than those of systemic chemotherapy and can be improved soon after symptomatic treatment. The main adverse reactions of lenvatinib are hypertension, proteinuria, and hypothyroidism [19]. Moreover, the most common adverse reactions of immunotherapy include fatigue, rash, pruritus, and diarrhea [57]. In this study, the main adverse reactions of HAIC combined with lenvatinib and PD-1 antibody were transaminase elevation, thrombocytopenia, albumin reduction, nausea and vomiting, leukopenia, abdominal pain, and hypertension. There were 7 patients (21.88%) with grade 3 or above adverse reactions, 2 patients (6.25%) with elevated transaminase, 1 patient with leukopenia, 1 patient with thrombocytopenia, 1 patient with nausea and vomiting, 1 patient with abdominal pain, and 1 patient with diarrhea. The dose of lenvatinib was reduced as elevated transaminase, leukopenia, thrombocytopenia, and diarrhea in five patients. The adverse reactions above grade 3 can be improved by prolonging the hospitalization time and giving symptomatic treatment. The safety of this combination therapy was management. The additional treatment of HAIC didn’t increase adverse events compared to standard treatment [13, 19, 21].

However, his study has the following limitations: First, this study is retrospective, and there may be some confounding factors that may affect the treatment efficacy; second, This study was a small sample study with only 32 cases included, and further studies with larger sample size are needed in the future; Third, 27 patients were alive at the end of the follow-up in this study, so the correlation analysis of overall survival was not performed in this study; Finally, no subgroup analysis of different types of PD-1 monoclonal antibody was performed, which may cause bias in the results and adverse reactions.

In conclusion, this study demonstrated that HAIC combined with lenvatinib and PD-1 inhibitor as the first-line treatment for HCC complicated with MaVI was effective with high objective remission rate and disease control rate. Moreover, conversion surgery might be benefit from the triple combination. extrahepatic metastases and Child-Pugh score were independent prognostic factors of progression-free survival. Furthermore, minority of the patients suffered grade 3 or above adverse events and adverse reactions are tolerable.

## Data Availability

The datasets used and/or analyzed in the present study are available from the corresponding author upon reasonable request.
